# Differential Diagnosis of Popliteal Artery Entrapment Syndrome in Light of an Atypical Clinical Manifestation

**DOI:** 10.7759/cureus.34889

**Published:** 2023-02-12

**Authors:** Jaafar Abou-Ghaida, Deekshya Thapaliya, Ignacio Rua

**Affiliations:** 1 Medical School, Nova Southeastern University Dr. Kiran C. Patel College of Osteopathic Medicine, Clearwater, USA; 2 Vascular Surgery, Baptist Health South Florida, Miami, USA

**Keywords:** peripheral artery disease, acute arterial popliteal occlusion, rich’s classification, paes, popliteal artery entrapment syndrome

## Abstract

Popliteal artery entrapment syndrome (PAES) is a type of arterial obstruction seen in athletic and young patients with no cardiovascular risk factors. It is caused by aberrant anatomy affecting the position of the popliteal artery or gastrocnemius muscle or functional obstruction resulting from a hypertrophied gastrocnemius muscle. Rich's classification has been used to define the various entities. PAES presents as unilateral claudication exacerbated by physical exertion. However, such a clinical presentation is shared amongst not only vascular diseases but also musculoskeletal diseases. Therefore, a wide array of differential diagnoses must be considered when popliteal artery entrapment-induced claudication is suspected.

## Introduction

Popliteal artery entrapment syndrome (PAES) is a rare form of peripheral arterial obstruction commonly seen in young, athletic male patients with no atherosclerosis risk factors who partake in sports such as running, dancing, and swimming [1-3]. PAES presents as unilateral calf claudication exacerbated during physical activity [1-3]. One systematic study mentions the mean age to be 32 years old with 83% of cases in males [4]. The diagnosis is difficult to make due to the many differentials and low clinical suspicion, especially in atypical cases. However, if a diagnosis is made and early surgical intervention is instituted, the prognosis can be favorable.

**Figure 1:**
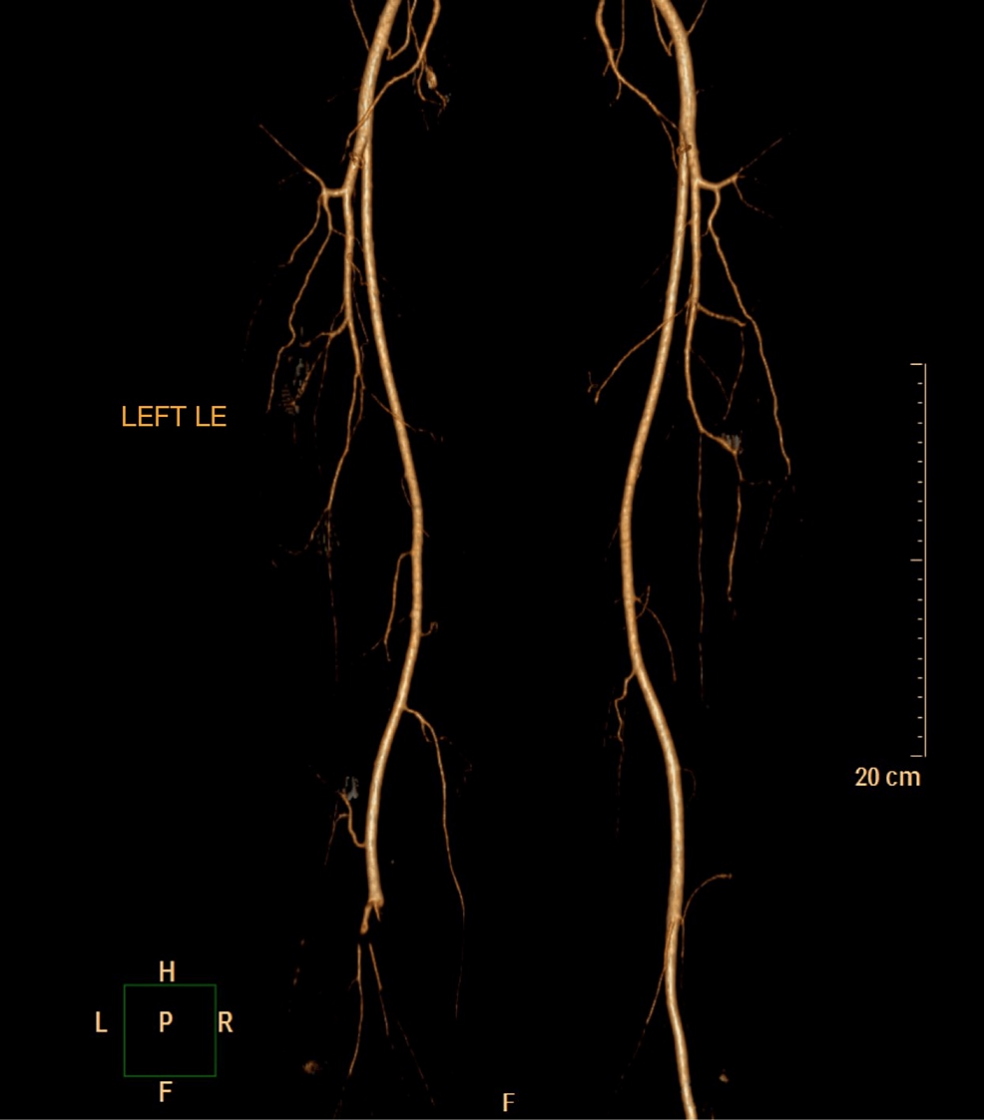
Computed tomography angiography (CTA) revealing left popliteal artery entrapment

The pathophysiology of PAES remains unclear. However, entrapment has been noted to be either due to aberrant anatomy or functional obstruction in the popliteal region. Normally, the popliteal artery is the deepest structure of the popliteal fossa running between the two heads of the gastrocnemius muscle before bifurcating into the anterior tibial artery and tibioperoneal trunk. In PAES, five anatomical variations, known as Rich’s classification, have been anatomically described (Figure 1). In type 1, the popliteal artery runs medial to the medial head of the gastrocnemius. In type 2, the medial head of the gastrocnemius attaches laterally to the popliteal artery. In type 3, both the popliteal artery and gastrocnemius are positioned normally, but an accessory muscle slip impinges on the popliteal artery. In type 4, the popliteal artery is obstructed by the popliteus muscle. Type F is functional obstruction involving a hypertrophied gastrocnemius muscle [1-3,5].

Even though the current literature portrays PAES as occurring most commonly in young, healthy, and athletic individuals, the presenting case involves a patient that does not perfectly fit the clinical picture in terms of age and risk factors. This may suggest that PAES can be acquired later in life possibly from strain or trauma affecting the normal anatomical position of the popliteal artery or embryological defects. Diagnostic tools are available to vascular surgeons which include digital subtraction angiography, computed tomography, ultrasonography, and magnetic resonance angiography [5]. Treatment options include botulinum toxin injections, surgical decompression, bypass grafting, and thromboendarterectomy [5].

## Case presentation

**Figure 2:**
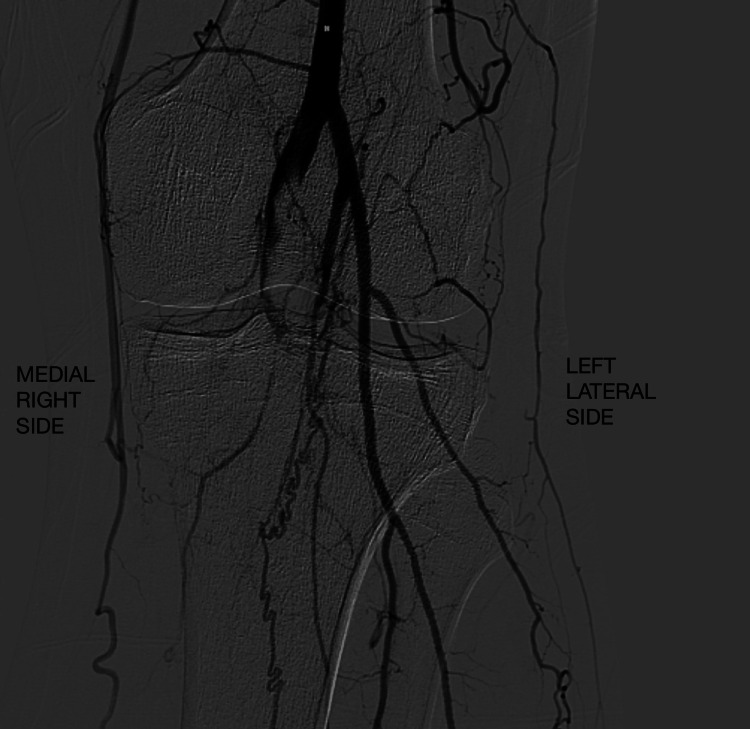
Left lower extremity digital subtraction angiography (DSA) indicating the entrapment of the left popliteal artery

The patient is a 46-year-old male who presented with progressive worsening left calf claudication that started three months ago, hindering his ability to exercise. The patient is a non-smoker, non-diabetic, with a history of hypertriglyceridemia. The patient denied fever, chills, chest pain, shortness of breath, dyspnea on exertion, and both upper and lower extremity weakness. The patient appeared alert and oriented to person, place, and time. A physical examination of the heart revealed no murmurs, heaves, or lifts. A physical examination of the lungs revealed no labored breathing, respiratory distress, or abnormal breath sounds. The abdomen was soft and non-tender with active bowel sounds. Left lower extremity examination revealed the left first and second toes along with the dorsal aspect of the left foot to be cool to the touch. In addition, the left calf was tender to palpation and the left dorsalis pedis pulse was nonpalpable. Capillary refill was greater than three seconds in the left lower extremity. However, there was no left extremity edema, with a normal range of motion of the left lower extremity. Examination of the right lower extremity revealed no clubbing, cyanosis, or edema. A computed tomography angiography (CTA) performed after hospitalization revealed popliteal artery occlusion extending to the tibioperoneal trunk with its proximal origin at the anterior tibial artery (Figure 1). In addition, angiography provided a guide for future procedures and confirmed previous findings (Figure 2). Subsequently, the patient was diagnosed with acute popliteal artery occlusion.

**Figure 3:**
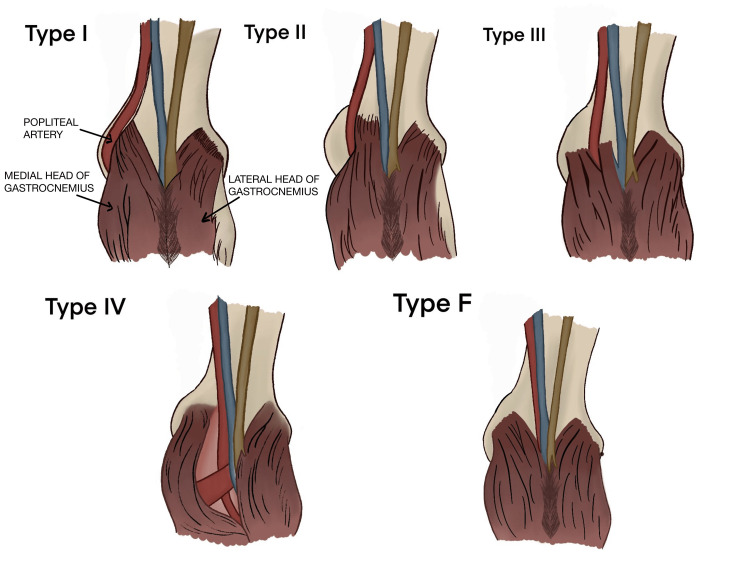
Rich's classification of the five variants of popliteal artery entrapment syndrome (PAES) Author's own illustration [6]

A heparin drip was started in order to deal with possible acute etiologies responsible for the patient’s presentation of thromboembolism. However, the lack of clinical improvement prompted the use of ultrasound-guided thrombolysis. However, that also failed to recanalize the patient’s circulation. The patient was then scheduled for surgical decompression. During surgery, a type 2 entrapment of the popliteal artery of the left leg was noted (Figure 3). Popliteal artery entrapment was relieved following the excision of the tendon of the medial head of the gastrocnemius which was inserted between the popliteal artery and vein. Additionally, during the surgical procedure, a selective embolectomy of the anterior tibial artery was performed which recovered multiple chronic thrombi. Furthermore, selective endarterectomies involving areas of atherosclerotic plaques were performed that resulted in re-establishing blood flow as demonstrated by a strong pulse extending to the distal two-thirds of the left lower extremity. Alternative options such as bypass surgery via saphenous vein grafting were not needed. Ankle-Brachial Index (ABI) obtained afterward revealed a value of 0.98 in the left lower leg, indicating the return of normal circulation and relief of the obstruction.

## Discussion

PAES is a form of peripheral arterial obstruction seen most commonly in young athletic patients without atherosclerotic risk factors. It is a challenging diagnosis due to several differentials with a similar presentation. Furthermore, such a diagnosis can be missed especially when a patient does not perfectly fit the agreed-upon clinical picture. Causal factors for PAES still remain unclear, with some studies suggesting that congenital malformations, genetic factors, or acquired mishaps may play a role [7]. Unfortunately, not enough research exists to identify a specific genetic defect or embryological malformation for PAES. Differential diagnoses include vascular and nonvascular etiologies. Vascular etiologies include peripheral arterial disease, cystic adventitial disease, and arterial endofibrosis. Non-vascular etiologies include chronic exertional compartment syndrome, tibial and fibular stress fracture, medial tibial stress syndrome, and referred pain from lumbar disc herniation [8,9].

Peripheral artery disease (PAD) was considered in this case. It is a vascular obstruction characterized by atherosclerosis with risk factors consisting of smoking, dyslipidemia, poor diabetic control, and hypertension [10]. PAD can cause vascular obstruction and may lead to critical limb ischemia or gangrenous changes. Patients may complain of intermittent claudication when active, relief of symptoms when resting, and a non-palpable distal pulse being an important clinical finding on examination [10]. Ankle-brachial index of less than 0.9 is diagnostic of PAD. Blood pressure measurements, echocardiograms, hemoglobin A1c, and lipid panels are investigative tools that can aid in clinical assessment [10].

Cystic adventitial disease is a nonatherosclerotic disease. Cyst formation occurs between the adventitia and tunica media of arteries, most often the popliteal artery [11]. Cysts may be unilocular or multilocular and may contain mucin. It presents more often in males in the fourth and fifth decade of life, and its exact etiology remains unclear. Its presentation is similar to PAES with the addition of Ishikawa’s sign or loss of pedal pulses with knee flexion. Diagnosis is typically made with duplex ultrasonography [11].

Arterial endofibrosis is a vascular disease afflicting primarily the external iliac artery. It presents mostly in cyclists as leg weakness and thigh pain on exertion [12]. Unlike PAD which constitutes the presence of atherosclerotic plaque, arterial endofibrosis is characterized by the buildup of loose connective tissue within the tunica intima of arterial walls [12]. The etiology or inciting events behind arterial endofibrosis still remains unknown. There is no standard to diagnose arterial endofibrosis. However, digital subtraction angiography allows the identification of the location of the stenotic area prior to surgical intervention. CT angiography may reveal the stenosis; however, some patients have been noted to have minimal arterial endofibrosis which was not identified on CT angiography [12].

Chronic exertional compartment syndrome (CECS) is an increase in pressure within a compartment of a limb, usually the anterior compartment of the lower leg. Patients most at risk for CECS are typically athletic individuals between 18-25 years old. However, it does have an equal predisposition to males and females. The increased pressure can compress arteries and nerves leading to pain, pallor, paralysis, pulselessness, and paresthesia. In addition, foot drop may occur during exercise [13]. More so, paresthesia is usually present in the anterior leg or between the first and second metatarsals due to the involvement of the medial terminal branch of the deep peroneal nerve [14]. Symptoms are relieved with rest and exacerbated with activity. Chronic exertional compartment syndrome is a diagnosis of exclusion and vascular etiologies must be evaluated first.

Stress fracture of the tibia and fibula usually occurs in athletes from a sudden increase in their activity level. Patients complain of a gradual increase in activity-related pain over several weeks to months. Eventually, the pain may worsen and can even occur with rest. There is focal tenderness and swelling around the fracture site. An inability to hop on a symptomatic leg for 10 repetitions without excessive pain constitutes a positive hop test and is strongly suggestive of a stress fracture [15]. Initial plain radiographs may not be sensitive enough to diagnose a stress fracture. However, an MRI can be done for earlier confirmation of a fracture. Medial tibial stress syndrome is characterized by pain in the tibia, especially the posterior medial tibial border. It usually occurs due to overuse which can commonly occur in athletes [16]. Initially, pain decreases with activity; the earlier onset of pain with more frequent training is seen in the later stages of the disease. On examination, provocative testing with resisted flexion is a positive finding. Radiological tests can be done to rule out a stress fracture.

Pain from PAES can sometimes be confused with referred pain from lumbar disc herniation. Pain in lumbar disc herniation is often sharp and shooting. It extends from the buttocks down into the back of the leg. The patient might complain of numbness and a tingling sensation in the leg and/or feet. Pain can commonly increase in intensity while in a seated position due to compression of the affected nerve. A straight leg test where the patient elevates the affected leg slowly, producing a reproduction of the patient's symptoms at an angle less than 45 degrees is a positive test. MRI is the gold standard for confirmation of lumbar disc herniation [17].

Adductor canal compression syndrome is an entrapment that occurs to the superficial femoral artery either in the adductor canal by fibrous bands arising from the adductor magnus muscle or by a hypertrophied vastus medialis or adductor magnus muscle, similar to PAES [18]. It presents as intermittent claudication in young physically active runners and skiers and can lead to limb-threatening ischemia [18]. Symptoms such as paleness, coldness, or weak peripheral pulses are rare, especially in the early stages of the disease, making it necessary to understand a patient's history [19].

Soleal Sling Syndrome is characterized by compression of the tibial nerve as it passes under the soleal sling. Patients present with plantar pain, numbness, and calf tightness or pain without intermittent claudication. It can be mistaken for tarsal tunnel syndrome. Characteristic physical examination findings include pain with palpation along the tibial nerve route as it passes under the soleal sling (positive Tinel sign) in the posterior knee as well as isolated weakness of the flexor hallucis longus [20].

Similarly to PAD, cystic adventitial disease, arterial endofibrosis, and PAES can present with signs of arterial occlusion (cool and pale extremity with diminished pulses). This stresses the need for a thorough history in order to be able to differentiate these diagnoses. Peripheral artery disease, on the one hand, is an atherosclerotic disease resulting from lifestyle choices and chronic illnesses such as smoking, hyperlipidemia, diabetes, and hypertension. PAES and cystic adventitial disease, on the other hand, can present in otherwise healthy individuals with none of the aforementioned risk factors as well as a history of sports involvement. However, cystic adventitial disease can be distinguished from PAES with Ishikawa’s sign and duplex ultrasonography revealing anechoic masses. PAES is best diagnosed with CTA and MRI which visualizes the aberrant location of the gastrocnemius muscle in relation to the popliteal artery. Non-vascular etiologies involving the musculoskeletal system are mostly found in healthy and athletic individuals. Therefore, a detailed history, including a patient’s exercise/sport involvement, history of trauma, a musculoskeletal physical workup, and imaging studies can prove very useful in differentiating these non-vascular diseases from their vascular counterparts.

## Conclusions

Popliteal artery entrapment syndrome is an obstructive disease requiring prompt reperfusion therapy to the affected limb to prevent ischemic damage. PAES can manifest suddenly and may not necessarily fit the typical clinical picture that various research articles have portrayed. Follow-up following vascular surgery revealed that our patient was ambulating well and recovery of his lower limb mobility with a pounding dorsalis pedis pulse had been achieved.
